# Computational Design of the β-Sheet Surface of a Red Fluorescent Protein Allows Control of Protein Oligomerization

**DOI:** 10.1371/journal.pone.0130582

**Published:** 2015-06-15

**Authors:** Timothy M. Wannier, Matthew M. Moore, Yun Mou, Stephen L. Mayo

**Affiliations:** 1 Division of Biology and Biological Engineering, California Institute of Technology, Pasadena, California, United States of America; 2 Division of Chemistry and Chemical Engineering, California Institute of Technology, Pasadena, California, United States of America; NCI-Frederick, UNITED STATES

## Abstract

Computational design has been used with mixed success for the design of protein surfaces, with directed evolution heretofore providing better practical solutions than explicit design. Directed evolution, however, requires a tractable high-throughput screen because the random nature of mutation does not enrich for desired traits. Here we demonstrate the successful design of the β-sheet surface of a red fluorescent protein (RFP), enabling control over its oligomerization. To isolate the problem of surface design, we created a hybrid RFP from DsRed and mCherry with a stabilized protein core that allows for monomerization without loss of fluorescence. We designed an explicit library for which 93 of 96 (97%) of the protein variants are soluble, stably fluorescent, and monomeric. RFPs are heavily used in biology, but are natively tetrameric, and creating RFP monomers has proven extremely difficult. We show that surface design and core engineering are separate problems in RFP development and that the next generation of RFP markers will depend on improved methods for core design.

## Introduction

Computational methods have heavily influenced protein design, but despite successes in core-repacking, computational design of protein surfaces, especially those with a high β-sheet content, has lagged [1]. There are relatively few instances of demonstrated success in β-sheet design [2–4]. Directed evolution, having proven effective at tackling problems that computational protein design (CPD) is ill-equipped to address, has been used to some success to evolve soluble β-sheet surfaces [5, 6]. Directed evolution, however, requires high-throughput screening, and is inefficient, as error-prone mutagenesis is used to randomly walk through sequence space. Here we present a CPD-driven library creation process that can efficiently search sequence space for soluble protein surfaces, facilitating surface design of proteins in situations that are not readily adaptable to high-throughput screening methods [7, 8]. We demonstrate the successful design of a fluorescent protein (FP) β-sheet surface, expediting monomerization of a core-stabilized RFP.

Oligomerization is a significant barrier to novel FP development. Most native FPs are oligomeric [9–11], and many engineered FPs that are thought to be monomeric exhibit dimerization in certain laboratory or biological contexts, complicating data interpretation, and even contributing to erroneous scientific findings [12, 13]. Soluble, monomeric FP probes are needed to prevent FP-driven aggregation or FP-mediated assembly of linked protein targets, and to limit the cytotoxic effects of poorly soluble proteins [12, 14]. A major challenge facing FP-engineering is breaking oligomeric interfaces without negatively impacting the fluorescent characteristics of a wild-type FP. To do so means designing soluble, β-barrel surfaces that are not aggregation prone. No standard technique has emerged for efficiently and effectively moving from a dimeric or tetrameric FP to a monomer without extensive intuition-based mutagenesis to disrupt oligomerization, followed by successive rounds of directed evolution to restore fluorescence [15].

The most challenging FPs to monomerize have proven to be red FPs (RFPs). All known native RFPs are tetrameric, the vast majority of which have not been extensively used or characterized because of the difficulty of breaking their oligomerization without compromising fluorescence. Of the more than 50 native RFPs described to date, only four have been successfully monomerized, as determined by a variety of *in vitro* methods, and in each case there has been significant mutation to the core of these proteins, often blue-shifting their fluorescent excitation and emission spectra and decreasing their brightness and photostability [5, 12, 16–18]. Efforts to improve brightness, engineer bathochromic shifts, or otherwise improve existing RFPs have focused on engineered monomers, and so have targeted only a small subset (< 10%) of known RFP biodiversity. The link between RFP monomerization and its negative impact on the spectroscopic properties of the resultant monomers is poorly understood.

Here we explore the engineering of monomeric RFPs, attempting to deconvolute the design of a soluble β-barrel surface from any impacts that core mutations have on an FP’s spectroscopic properties. We show that CPD, used in conjunction with efficient library construction and screening, is an effective tool to engineer the surface of an RFP-type β-barrel protein. We tested our method in a hybrid protein engineered as a cross between DsRed, a native tetrameric RFP, and mCherry, a monomeric variant of DsRed, from which we took the evolved protein core (13 mutations from DsRed). Screening a small library of computationally designed variants of a tetrameric RFP, we found that 97% were bright, stable, monomeric, and little changed spectroscopically. This process represents a significant improvement to the speed and efficiency of RFP monomerization, and may facilitate the study of a much broader array of native RFPs, allowing researchers to target engineering efforts to residues in the protein core, as structural stabilization of the chromophore environment appears to be the primary bottleneck to RFP monomerization. This computationally driven method for the monomerization of fluorescent proteins should be applicable as a general technique for creating soluble monomeric protein variants.

## Materials and Methods

### Plasmids and Bacterial Strains

DsRed and mCherry sequences were taken from their Genbank entries (accession numbers AF168419 and AY678264). Ten amino acids were added to the N-terminus of each protein, consisting of a methionine followed by a 6x histidine tag for protein purification, and then followed by a Gly-Ser-Gly linker sequence. All gene sequences were constructed with gene assembly PCR, oligonucleotides for the assembly were designed with DNAworks, and then ordered from Integrated DNA Technologies (IDT). Assembled genes were PCR-amplified and cloned into the pET-53-DEST expression plasmid (EMD Millipore) with PIPE cloning followed by CPEC. Constructs were sequence-verified (Laragen) with a primer specific to the T7 promoter. BL21-Gold(DE3) competent cells, a protein expression strain (Agilent), were then transformed with the sequence-verified plasmid constructs.

### Construction of Designed Libraries and Variants

Explicitly-designed DsRmCh, mLib, and rLib protein sequences were input into DNAworks as “mutant runs” of the wild-type DsRed gene assembly. This allows explicit libraries of gene variants to be assembled and minimizes the number of oligonucleotides needed. Oligonucleotides were ordered from IDT and cloning was carried out as described above.

### Protein Expression and Purification

Single bacterial colonies were picked with sterile toothpicks and inoculated into 300 μl of Super Optimal Broth (SOB) supplemented with 100 μg/ml ampicillin in 2 ml deep-well 96-well plates (Seahorse Bioscience). The plates were sealed with microporous film to facilitate gas exchange during growth. Cultures were grown overnight at 37°C / 300 RPM. The next morning 800 μl of fresh SOB with 100 μg/ml ampicillin and 1 mM Isopropyl β-D-1-thiogalactopyranoside (IPTG) was added to a total volume of 1 ml (evaporation losses overnight were approximately 100 μl). Plates were then shaken 12 hours at 37°C / 400 RPM. Cell cultures turn red if there is strong RFP expression. The 96-well plates were then centrifuged at 3,000 x g in a swinging-bucket rotor and the supernatant was decanted. Pellets were resuspended in 500 μl of lysis buffer (50 mM sodium phosphate, 150 mM NaCl, 0.1% v/v Triton-X, 10% v/v 10x Cell Lytic B, pH 7.4) supplemented with 50 Units/ml Benzonase and 0.05 mg/ml Hen Egg Lysozyme. Plates were then shaken on a benchtop plate shaker with a 3 mm orbital stroke length (Heidolph) at 1,000 RPM for 30 minutes. To pellet the cellular debris, the plates were again centrifuged for 10 minutes at 3,000 RPM in a swinging-bucket rotor. The colored supernatant was then applied to a 96-well His-Select filter plate (Sigma), washed twice (50 mM sodium phosphate, 150 mM NaCl, 15 mM Imidazole, pH 7.4), and eluted with 500 μl elution buffer (50 mM sodium phosphate, 150 mM NaCl, 250 mM Imidazole, pH 7.4). All His-Select purification steps were performed at 1,000 x g in a swinging bucket rotor.

### Fluorescent Protein Characterization

Purified proteins were assayed in triplicate in Greiner UV-Star 96-well plates with a Tecan Safire2. An absorbance scan (260–650 nm), a fluorescence excitation scan (500–640 nm excitation / 675 nm emission), and a fluorescence emission scan (550 nm excitation / 575–800 nm emission) were run on 100 μl of eluted protein to determine spectral peaks.

#### Quantum Yield

To measure quantum yield, we diluted each protein so that the absorbance for 200 μl of protein at 540 nm was between 0.1 and 0.5. We then measured the A_550_ in triplicate (or duplicate for poorly expressed proteins), diluted the sample to an A_550_ of 0.04 and collected emission spectra (540 nm excitation / 550–800 nm emission). The area under the emission curve was calculated after fitting to a 4^th^ order Gaussian, and the quantum yield was calculated with the following formula:

$$\Phi_{x}=\left(\frac{A_{s}}{A_{x}}\right)*\left(\frac{F_{x}}{F_{s}}\right)*{\left(\frac{n_{x}}{n_{s}}\right)}^{2}*\Phi_{s}$$ (1)

Where Φ is quantum yield, A is absorbance, F is total fluorescent emission (area under the curve), and n is the refractive index of the solvents used. Subscript “X” refers to the queried substance and subscript “S” refers to a standard of known quantum yield. It is important that the standard be excited with the same wavelength of light as the unknown sample. We use DsRed, which has a known quantum yield of 0.79 as the protein standard.

#### Extinction Coefficient

To measure extinction coefficient we used 100 μl of the protein solution that had been diluted to an A_550_ of between 0.1 and 0.5 and measured absorbance between 400 nm and 700 nm in triplicate. We then added 100 μl of 2M NaOH to each well and remeasured absorbance between 400 nm and 700 nm. The base-denatured chromophore, which has an absorption peak maximum at approximately 450 nm, has a known extinction coefficient of 44,000 M^-1^cm^-1^. The extinction coefficient is calculated with the following formula:

$$\varepsilon \;=\;A_{Chromophore}*44,000M^{-1}cm^{-1}/A_{450}$$ (2)

To measure thermal stability, purified proteins were diluted to an absorbance of 0.2 at the wavelength of maximum absorbance (λ_abs_) so that their fluorescence would not saturate the detector. 50 μl of each purified protein was then loaded into a 96-well PCR plate and covered with clear optical tape. The proteins were incubated at 37°C for 10 minutes and then the temperature was ramped at 0.5°C every 30 seconds up to 99°C, with fluorescence measured every ramp step in a CFX96 Touch Real-Time PCR Detection System (Bio-Rad). We refer to this as a thermal melt. The derivative curve of the thermal melt finds the inflection point of the slope, which is the apparent temperature at which fluorescence is irrecoverably lost (apparent T_m_).

### Determination of protein oligomericity

#### Size Exclusion Chromatography

100 μl of purified protein was run over a Superdex 75 10/300 size exclusion column with 25 ml bed volume on a GE Life Sciences AKTA. Absorbance was measured after passage through the column at 575 nm.

#### Analytical ultracentrifugation

Protein samples were purified by SEC into PBS pH 7.4, and then diluted to an A_575_ of 0.5 for a path-length of 1.25 cm. These samples were put into two-channel sedimentation velocity cuvettes with the blank channel containing PBS. Sedimentation velocity was run at 40,000 RPM overnight with full A_575_ scans collected with no pause between reads. Data were loaded into Sedfit and a c(m) distribution was run with default assumptions made for PBS buffer viscosity. After integration, the c(m) curve was exported to Excel.

#### Homo-FRET

200 μl of purified protein in elution buffer was diluted with PBS pH 8.0 to an Absorbance of between 0.1 and 0.5 at 530 nm in 96-well Greiner UV-Star plates. Polarization scans were then taken with excitation at 530 nm and emission at 610 nm in a Tecan Safire2 plate-reader. Rose Bengal was used as a standard to calculate the instrument G factor (mP = 349).

### Computational Design Methods

The structure of DsRed was taken from a published high resolution crystal structure (PDB ID: 1ZGO) [19]. Waters and other non-protein atoms and molecules were removed in addition to three of the four protein chains present in the tetrameric crystallographic unit. Hydrogen atoms were then added to the remaining protein chain (chain A), which was then run through 50 steps of all-atom energy minimization to reduce strain and eliminate clashes due to van der Waals interactions. Single state design was run with the PHOENIX force field [20, 21], allowing mutations at 10 positions from the AC interface and 7 from the AB interface that were fully or partially buried. The FASTER algorithm [22] was used to sample a backbone dependent rotamer library [23]. We allowed the wild-type amino acid at each position in addition to the following amino acids: Ala, Arg, Asn, Asp, Gln, Glu, Gly, His, Lys, Pro, Ser, and Thr. The 96 variants with the lowest computed energies were selected for mLib.

## Results and Discussion

### Hybrid RFP Demonstrates the Functional Distinction Between Core and Surface Domains

**Fig 1:**
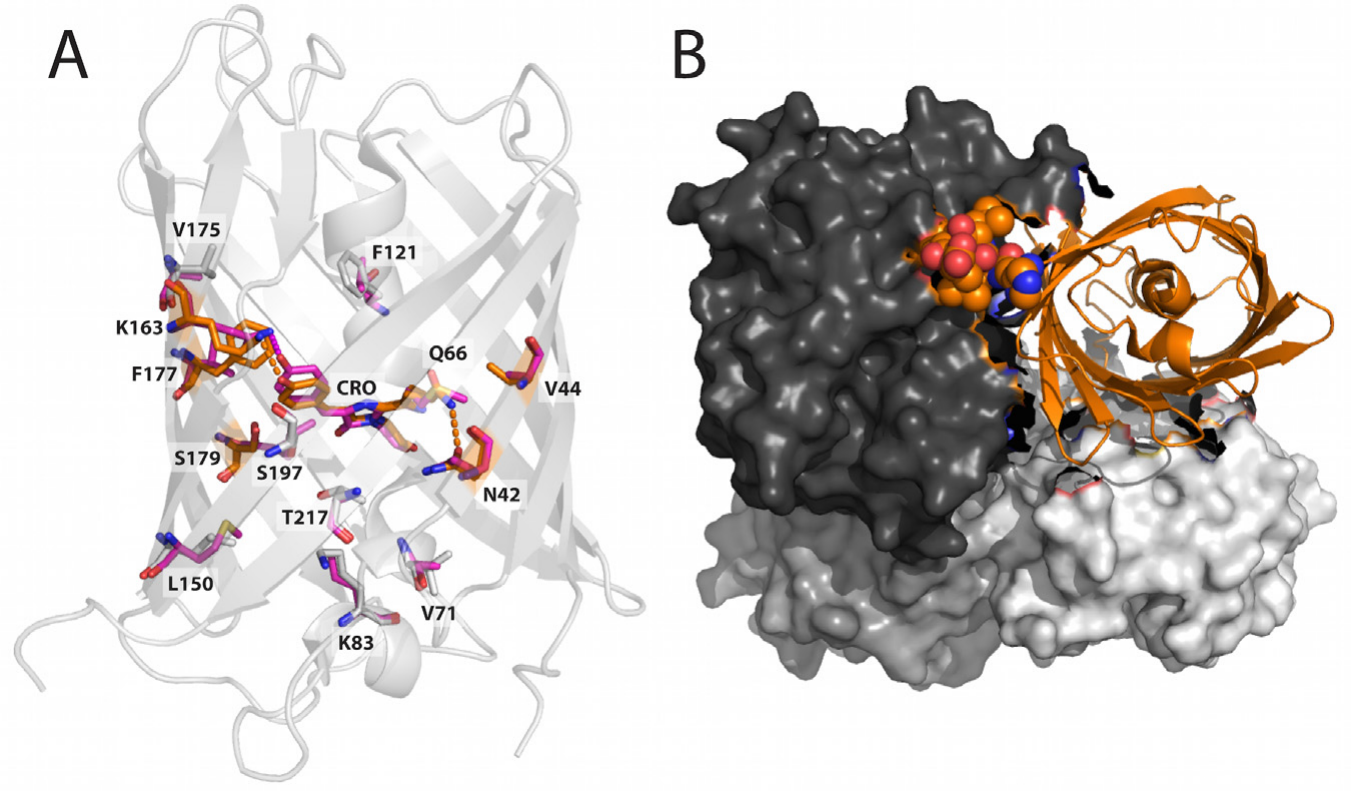
Structure of DsRed (PDB ID: 1ZGO). (A) Positions mutated in the core of the protein during the directed evolution of mCherry. DsRed is shown in light gray ribbon, mutated residues are shown in sticks, and those within 5 Å of the chromophore (CRO) are highlighted in orange. The aligned residues from mCherry (PDB ID: 2H5Q) are overlaid in pink. (B) DsRed chains B, C, and D are shown as light gray, dark gray, and gray surfaces respectively, while chain A is shown as an orange ribbon. The C-terminal tail of chain A, shown as spheres, stabilizes the AC interface between chain A and chain C. Below chain A in the image is chain B, with which chain A forms the AB dimeric interface.

**Fig 2:**
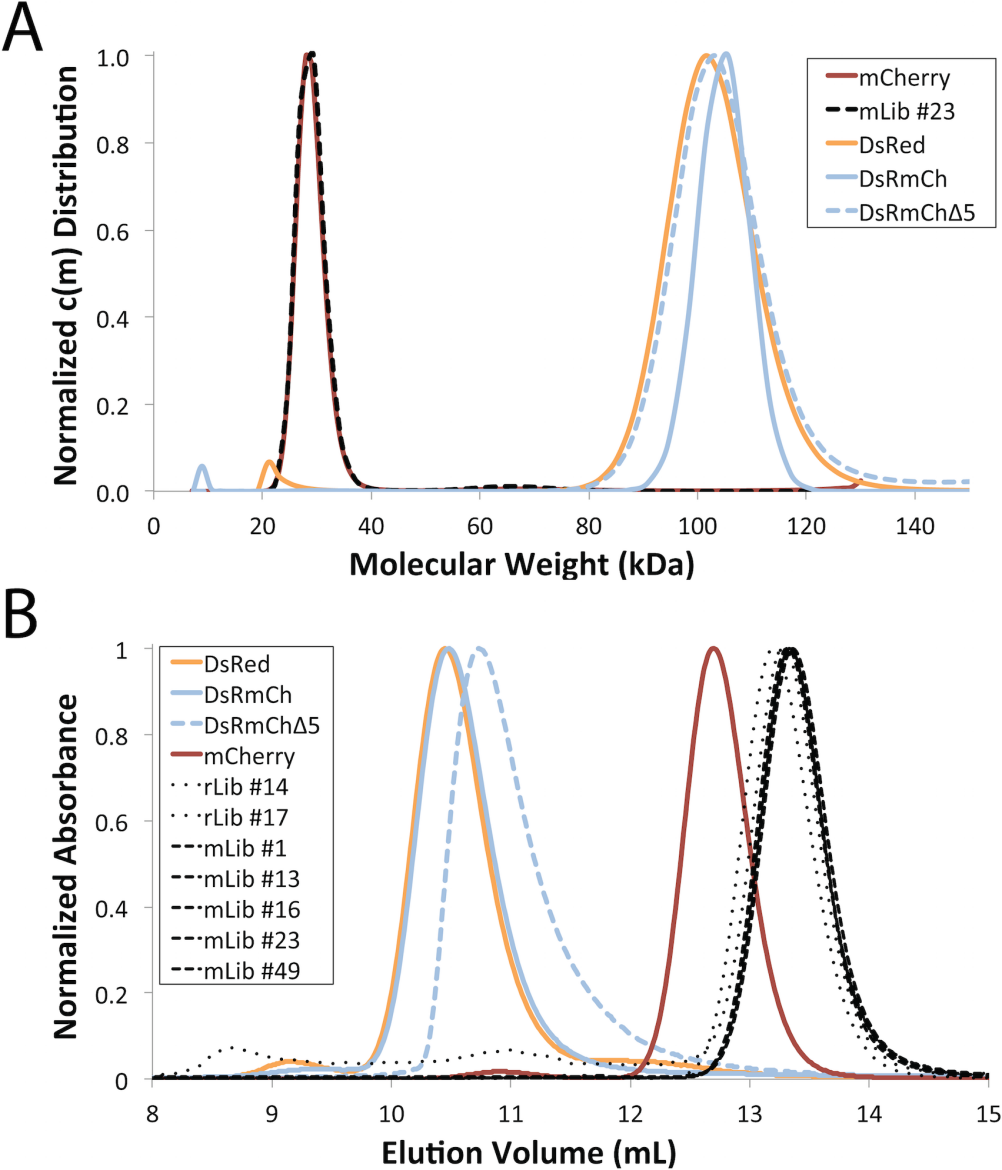
Low Throughput Oligomerization State Analysis of FPs by AUC and SEC. (*A*) c(m) distribution from AUC sedimentation velocity experiments. (*B*) Oligomerization state of FP variants analyzed by SEC (Superdex 75). In both A and B, the experiments are normalized to 1 for clarity of presentation.

To validate the computational design of β-barrel surfaces, we selected DsRed, as it has been twice independently monomerized via directed evolution and there exist >60 characterized monomeric variants of the protein. We set out to create a DsRed mutant that would tolerate surface mutations—possessing a structurally sound core that would retain fluorescence upon monomerization. Such a variant would allow us to separate the problem of surface design from the effects of mutations to the chromophore environment. We hypothesized that stabilizing mutations to the core of DsRed were both responsible for changes to its spectroscopic properties and necessary for monomerization, as no monomerization of a native RFP has been successful without altering the protein core [5, 12, 16–18]. One of the most studied and thoroughly characterized monomeric variants of DsRed is mCherry, a less-bright but red-shifted 30-point mutant [24]. We created a hybrid RFP (DsRmCh) that is a 13-point mutant of DsRed, containing every mutation to a residue in the core of the protein that was introduced during the evolution of mCherry (Fig 1). Consistent with the hypothesis that core residues are determinant of the fluorescent properties of an FP, DsRmCh is spectroscopically mCherry-like, but remains tetrameric. Specifically, DsRmCh retains the bathochromic shift to its fluorescent emission, decreased brightness, and accelerated maturation of mCherry, suggesting that the residues responsible for these properties are indeed among the 13 core mutations (Table 1). We measured the oligomerization state of DsRmCh, which retains a wild-type DsRed surface, by analytical ultracentrifugation (AUC) and size exclusion chromatography (SEC) (Fig 2). DsRmCh remains tetrameric indicating that the core residues are not implicated in oligomerization. Conversely, the inverse of DsRmCh, mChDsR, comprising an mCherry surface and a DsRed core, is not solubly expressed nor is it fluorescent (data not shown), indicating that stabilization to the core of DsRed is required to successfully monomerize the protein. To directly measure the stabilizing effect of DsRmCh’s core mutations we measured the apparent T_m_ of DsRed, DsRmCh, and mCherry (Table 1 and S1 Fig). DsRmCh is thermostabilized by 4°C relative to DsRed and 9°C relative to mCherry. Thus, the mutations that optimize DsRed’s core for monomerization are also thermostabilizing.

**Table 1:** Properties of DsRed, mCherry, DsRmCh, and Selected Variants.

RFP Name	# Non-tail mutations	C-terminal tail mutations (Δ/+ tail length)†	Excitation Max, or λ_ex_ (nm)	Emission Max, or λ_em_ (nm)	Quantum Yield, or Φ	Extinction Coefficient, or ε (M^-1^ cm^-1^ / 1000)	Brightness(Φ x ε / 1000)	Apparent T_m_ (°C)	Fluorescence in Culture‡ (% mCherry)
DsRed	—	—	558	585	0.79	73	58	94.5	—
DsRedΔ1	—	Δ1	558	584	0.57	53	30	84.0	—
mCherry	30	+6	588	611	0.22	85	19	89.5	100
mCherryΔ6	30	—	588	611	0.21	91	19	89.5	103
mCherryΔ11	30	Δ5	588	612	0.20	78	16	87.5	98
DsRmCh	13	—	585	611	0.23	97	22	98.5	133
DsRmChΔ5	13	Δ5	586	612	0.20	92	21	89.5	113
mLib Avg	26	Δ5	585	608	0.23	62	14	—	—
mLib Top	—	—	586	609	0.26	72	19	—	—
mLIb77	27	Δ5	586	609	0.23	72	17	89.0	72
mLib77 + DsRed Tail	27	—	584	609	0.23	72	17	91.5	45
mLib77 + mCherry Tail	27	+6	586	609	0.24	72	17	93.0	107

Spectroscopic properties of RFP variants. “mLib Avg” is the mean of mLib members;, “mLib Top” is the largest value of each attribute seen in mLib. “mLib Top” does not refer to a single, specific variant.

^†^ “Δ” indicates number of residues deleted from the C-terminal tail; “+” indicates number of residues added to the C-terminal tail.

^‡^ Fluorescence in culture was measured with the following wavelengths: 570 nm excitation and 610 nm emission.

### DsRmCh Monomerization with CPD

Having determined that mutations to the core of mCherry are responsible for the spectroscopic alterations to the protein, we next sought to determine if the optimized mCherry core would indeed facilitate monomerization. As an initial test of the robustness of the fluorescence in DsRmCh to surface perturbation, we partially destabilized the AC interface, the more stable of the two oligomeric interfaces, by deleting the protein’s five-residue C-terminal tail. The C-terminal tail stabilizes the AC interface via intermolecular interactions (Fig 1B), and deletion of even the two C-terminal most residues in DsRed completely abolishes fluorescence (data not shown). DsRmCh, however, tolerates a complete deletion of its C-terminal tail (residues 221–225). This variant, which we call DsRmChΔ5, however, remains tetrameric (Fig 2A) and preserves the spectroscopic properties of DsRmCh (Table 1). DsRmChΔ5 is less thermostable than DsRmCh (Table 1) and shows an altered SEC elution profile (Fig 2B), suggesting that the oligomeric interaction is destabilized. As DsRmCh was mostly unperturbed by deletion of its C-terminal tail, we thought that it would be an easier template for monomerization, as it was missing part of its AC interface. We set out to design, via CPD, the β-sheet surfaces of DsRmChΔ5 to fully monomerize the protein.

**Fig 3:**
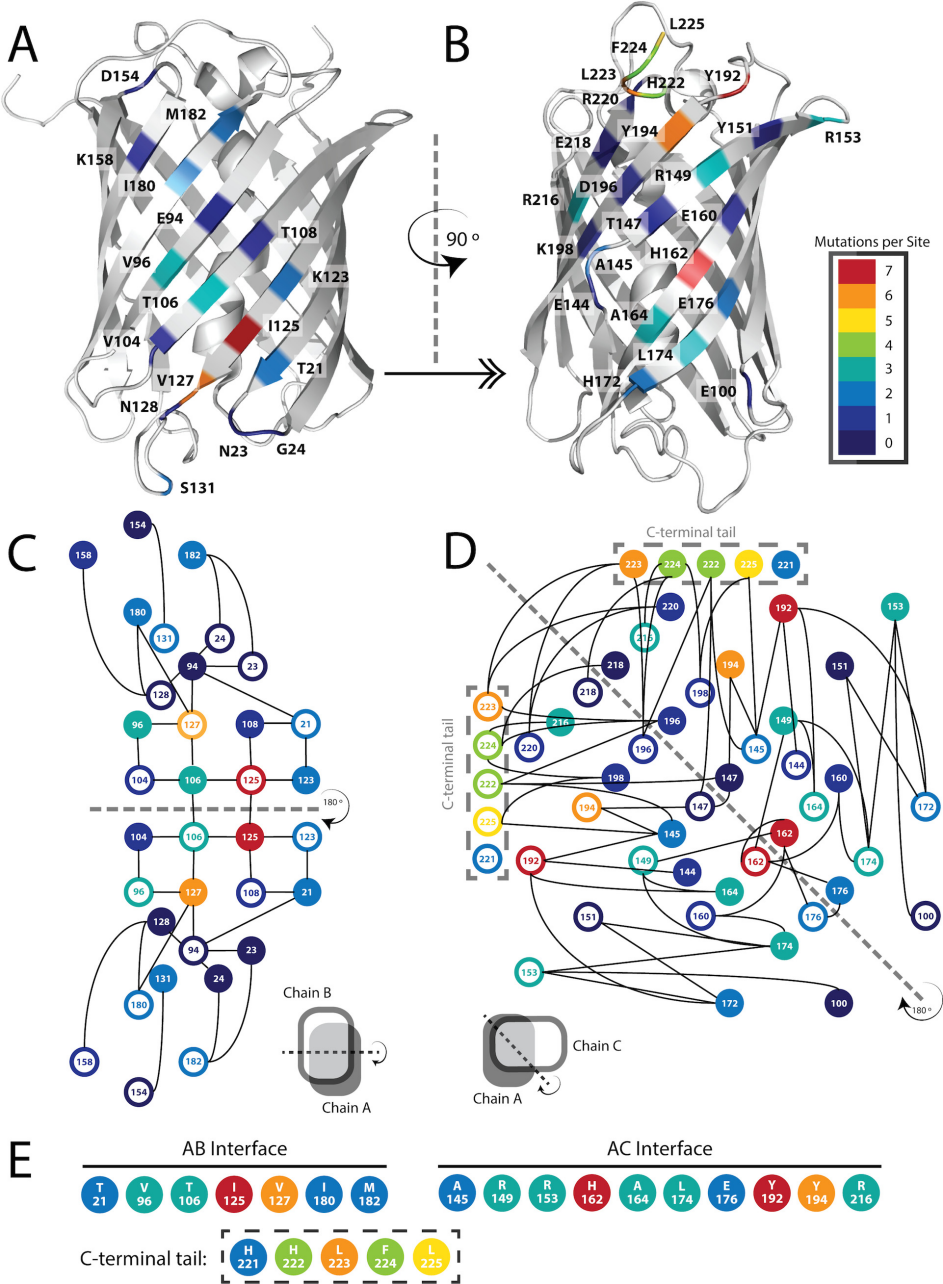
Schematic Representation of DsRed AB and AC Interfaces. (*A and B*) Color-coded representation of mutational frequency during the monomerization of a select group of FP monomers (mRFP1, DsRed.M1, mTFP1, mAG, mKO, mEosFP, mKeima, FusionRed, mRuby, Dendra, and Dronpa) mapped onto chain A of the crystal structure of DsRed (PDB ID: 1ZGO), showing the AB (A) and AC (B) interfaces. (*C and D*) A representation of the two interfaces showing inter-chain contacts with solid circles denoting residues from chain A and open circles denoting residues from the interacting chain (chain B and chain C, respectively). Black lines represent inter-chain oligomeric interactions and the dashed lines are lines of symmetry. Note that β-sheets from opposing subunits of the AB interface are stacked in an anti-parallel fashion, while those of the AC interface are offset by ~90° as represented by cartoon images below each interaction diagram. Note also that attention is called to the deletion of residues from position 221 to the C-terminus (C-terminal tail), which decreases the complexity of the AC interface. (*E*) Residues in DsRed selected for computational design in mLib including the C-terminal tail deletion.

**Fig 4:**
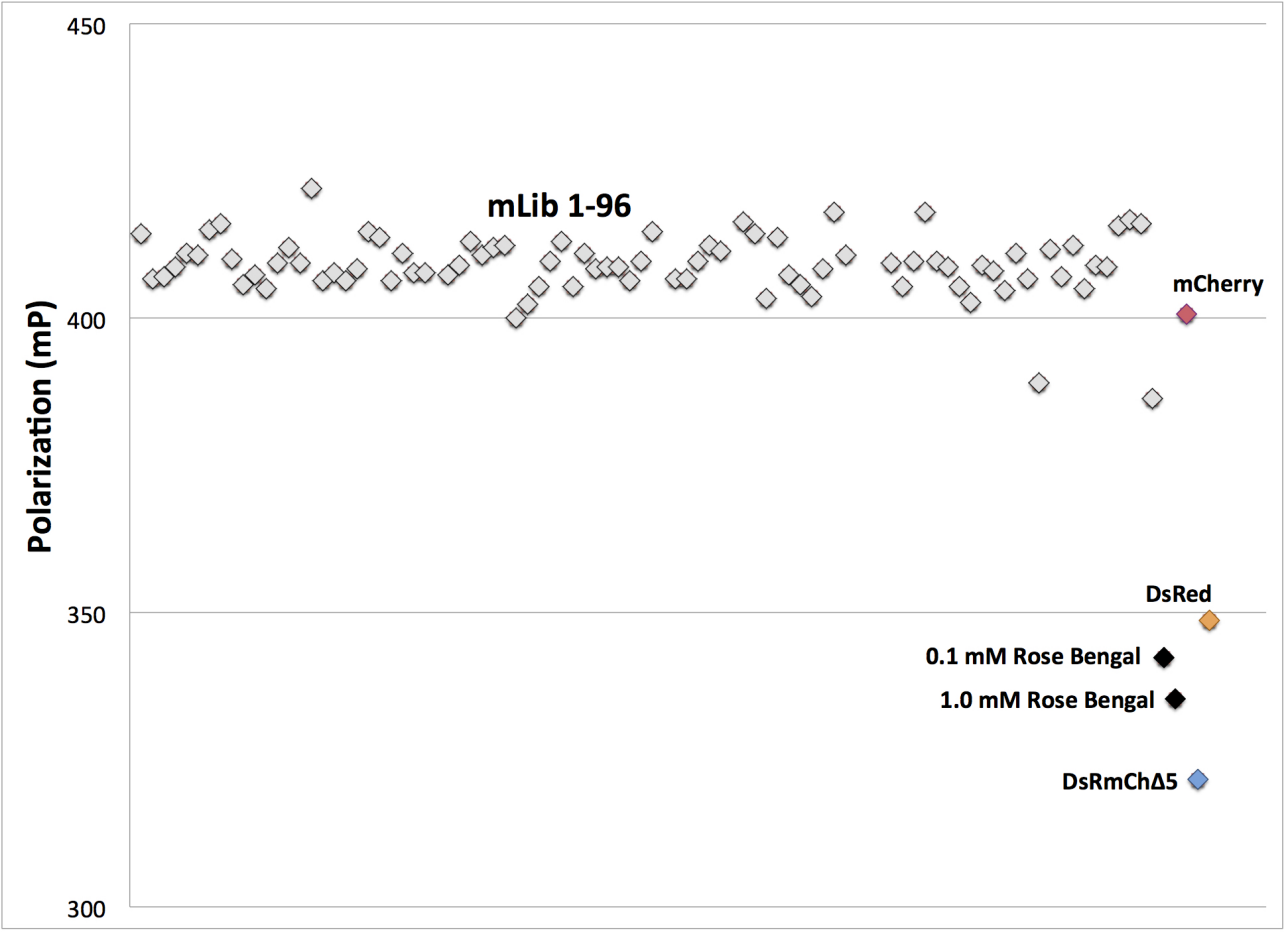
High Throughput Oligomerization State Analysis of mLib by Homo-FRET. Grey, 96 members of mLib; red, mCherry monomer control; yellow, DsRed tetramer control; blue, DsRmCh; black, Rose Bengal. Position along the x-axis serves only to separate individual variants for better visibility.

**Fig 5:**
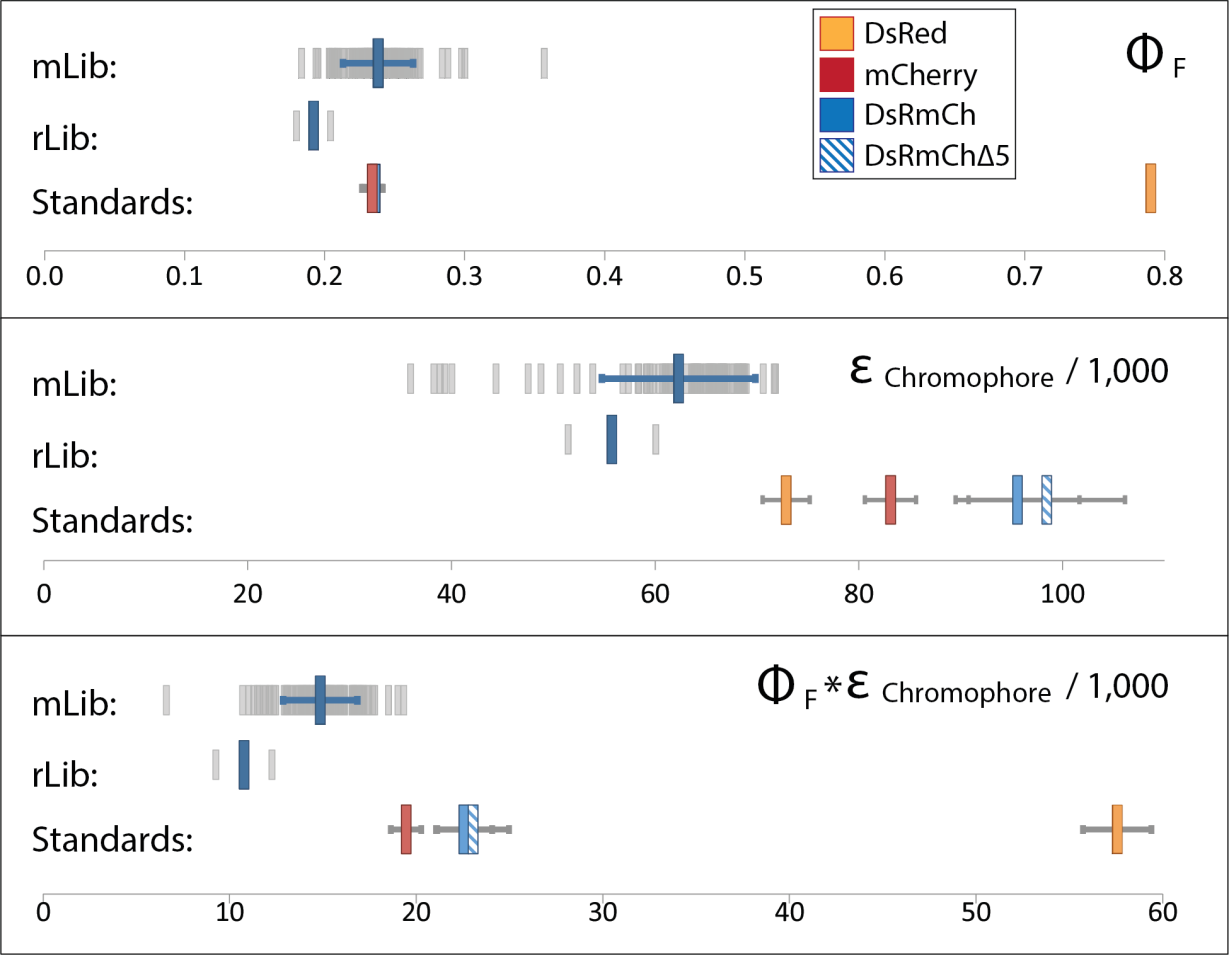
Characterization of Designed Libraries. Graphical representation of quantum yield (*Top*), extinction coefficient (*Middle*), and brightness (*Bottom*) of mLib, rLib, and controls. For mLib and rLib, light gray bars indicate individual variants, the larger blue bar represents the library mean, and blue horizontal bars represent one standard deviation from the mean. For standards, a legend is shown in the top chart, and error bars represent the standard deviation from three independent measurements.

In order to determine which residues to target for design, we analyzed DsRed’s two oligomeric interfaces, the AB and AC interface (Fig 1), which are named for the crystallographic chain names from the original structure of DsRed [25]. To narrow down the choices, we found eleven instances of FP monomerization in the literature, and made an alignment of the native FPs and their engineered monomeric variants [5, 12, 15, 17, 18, 26–31] (S2 Fig). We found 17 positions at the two interfaces that were largely buried, made significant intermolecular contacts, and that had been frequently mutated during the monomerization of previous FPs (Fig 3). We then targeted these positions, seven for the AB interface and ten for the AC interface, for surface design using CPD, and allowed the software to sample the wild-type amino acid and 12 additional amino acids at each position: Ala, Arg, Asn, Asp, Gln, Glu, Gly, His, Lys, Pro, Ser, and Thr. Consistent with the work of Kuhlman and colleagues, we did not need to use an explicit negative design to achieve a soluble β-sheet surface [4]. We constructed a monomeric library (mLib), which comprised the 96 designed variants with the lowest computed energy (S3 Fig). We then expressed and characterized mLib, and found 97% (93/96) of the mLib variants to be measurably fluorescent. As an initial evaluation of monomerization, the oligomerization state of the mLib variants was tested using a high-throughput homo-FRET assay, which measures the loss of polarization due to non-radiative energy transfer between neighboring chromophores (Fig 4). Every fluorescent member of mLib was also monomeric. To confirm the results of the homo-FRET assay, select members of mLib were shown by SEC and AUC to be monomeric (Fig 2). The members of mLib were significantly mutated, with 13–16 surface mutations per variant, but did not share significant mutational similarity to mCherry apart from the shared core mutations (S3 Fig). Nine of the 17 non-core sites that were mutated during the evolution of mCherry were targeted for mutation in mLib, but in only one instance was the amino acid residue present in mCherry selected by the CPD calculation. As mCherry and mLib variants shared the same protein core, the excitation and emission spectra of mLib variants were highly similar to those of mCherry (data not shown), and the mean brightness of the library was near that of the heavily evolved mCherry (Fig 5 and Table 1). Interestingly, the extinction coefficient of the average mLib member was less than that of mCherry, while the quantum yield of the average mLib member was greater than that of mCherry. We do not know the underlying biophysical reason for this variation.

In order to assess the effectiveness of the computational design process, we created a 24-member random control library (rLib), in which we allowed mutation at the same 17 positions as were designed in mLib. We randomly sampled the same set of amino acids that were allowed in the mLib design (12 amino acids plus wild type), but weighted their frequency of occurrence by their prevalence on the surface of bacterial mesophilic intracellular proteins [32]. We then characterized rLib, and when compared with mLib which was 97% fluorescent, we found only 8% (2/24) of rLib variants to be sufficiently fluorescent for accurate measurement. Most of the rLib variants expressed poorly (data not shown). Additionally, the two fluorescent variants that expressed well were both significantly dimmer than the median mLib variant (Fig 5).

In mLib we replicated the monomerization of mCherry, reproducing with one small, explicitly designed library, the surface optimization that was conducted over numerous successive rounds of evolution by fluorescence-activated cell sorting (FACS) and colony screening [24]. As an important confirmation of the value added by CPD, we showed that a control library, rLib, in which we attempted to break the interface with random surface mutation, was much less effective than the CPD-designed mLib. mLib’s CPD-designed surface mutations differ significantly from those isolated through the directed evolution of mCherry. This result is not surprising to us, as the potential sequence space available to a given fold is large, and thus disparate methods should not be expected to necessarily arrive at the same solution to a protein design problem.

### C-terminal Tail Effects

It has been proposed that a well-designed C-terminal tail is important to a monomeric FP, despite little supporting structural evidence [24]. We find that the C-terminal tail plays a critical structural role in obligate oligomers, but not in monomeric RFPs. As fluorescence was abrogated in DsRed but not in DsRmCh upon deletion of the C-terminal tail residues (residues 221–225), DsRmCh showed itself to be better able to tolerate monomerization. The evolved monomeric mCherry core presumably makes oligomerization unnecessary to DsRmCh fluorescence; hence the presence of a C-terminal tail has little impact. DsRed, by contrast, being maladapted to monomerization, loses significant fluorescence with the deletion of just one C-terminal residue, Leu225, and completely loses fluorescence with the deletion of a second (Table 1).

We observed, however, that some mLib variants, although spectroscopically similar to mCherry when characterized *in vitro*, did not express as robustly in culture. We reasoned that the C-terminal tail may have some effect on soluble protein expression. To gauge protein expression, we directly measured the fluorescence of induced bacterial cultures. We then chose four variants from mLib and added back a DsRed or an mCherry C-terminal tail to see if these tails would aid protein expression. Adding DsRed’s hydrophobic C-terminal tail (HHLFL) generally worsened expression while adding mCherry’s tail, which is a GFP mimic (HSTGGMDELYK) improved expression to a level equivalent to that of mCherry (S4 Fig). A culture expressing a representative variant of mLib, mLib77, was about 70% as bright as mCherry, but when mCherry’s 11-residue C-terminal tail was added to the protein, it expressed slightly better than mCherry and was more thermostable (Table 1).

We thus find through the study of mLib variants that a properly engineered C-terminal tail can improve the expression of monomeric RFPs. Furthermore, the addition of either the DsRed or the mCherry C-terminal tail to mLib variants generally improves their thermostability (S1 Fig), although neither C-terminal tail modification has any significant impact on brightness. We propose that a well-engineered C-terminal tail may help to prevent protein aggregation in some monomeric RFPs, but that further optimization of the protein may obviate the need for the tail altogether.

### Exploring the Core Mutations Found in mCherry

To further assess the degree to which core stabilization was necessary in the engineering of mCherry, and to better understand why mChDsR is not fluorescent, we conducted a series of mutational analyses of mCherry. First we reverted each of the 13 positions that had been mutated in the core of mCherry (and which were mutated in DsRed to make DsRmCh) to the wild-type residue found in DsRed. Ten of these mutants were detectably fluorescent, eight of which were about equally fluorescent to mCherry (Table 2). All ten mutants showed similar excitation and emission peaks, shifted only by ~2–3 nm. One reversion mutation of note was from a glutamine to a lysine at position 163, which improved mCherry’s quantum yield (Φ) from 0.22 and 0.24. We named this variant mCherryQ163K. In DsRed and mCherry, residue 163 makes van der Waals contact with the chromophore’s phenolate group, but the glutamine in mCherry appears to disrupt a hydrogen bonded water molecule that lysine in DsRed stabilizes along with the backbone carbonyl of residue 144 (DsRed: 1ZGO; mCherry: 2H5Q). Using mCherryQ163K as a template, we then continued to revert core positions to their wild-type DsRed residues, beginning with the highest quantum yield single reversion variants we had characterized. No reversion mutations to mCherryQ163K were found to be beneficial, and as successive core reversions were accumulated, even when the single revertants were minimally disruptive, the resulting proteins showed a steady loss of soluble expression and brightness. After several rounds of mutation and screening, we found a minimally mutated mCherry core that contained only seven mutations from the wild-type DsRed core compared to the 13 present in mCherry. This protein, which we call mCherryR6, has six core residue reversion mutations: A44V, A71V, L124F, M150L, Q163K, and T179S. Any further reversion mutations to the remaining seven mutated core sites of mCherryR6 resulted in a protein that was too weakly expressed to characterize. Thermostability measurements of these variants help to explain mCherry’s tolerance for the six core reversion mutations found in mCherryR6. The reversion mutation A217T had been the most puzzling, as it slightly improves mCherry’s brightness, but was not tolerated in the mCherryR6 background. Thermostability data, however, showed that this mutation lowers mCherry’s apparent T_m_ by 8.0°C, compared to a maximum of 3.5°C (seen in mCherry variant A44V) for the six mutations present in mCherryR6.

**Table 2:** mCherry Core Reversion Variants.

Mutation	Brightnes (% mCherry)	Apparent Tm (% mCherry)	Shift in emission (nm)
WT mCherry	100	100	—
A44V	93	96	+2
M68Q	90	95	-3
A71V	100	100	-3
L124F	99	99	—
M150L	91	98	-1
Q163K	108	98	—
A175V	84	98	—
V177F	24	93	+4
T179S	95	98	+1
A217T	104	91	-2
M150L / Q163K	104	99	-2
Q163K / T179S	94	99	+1
A71V / Q163K / T179S	100	99	-1
A71V / M150L / Q163K / T179S	95	98	-3
A71V / L124F / M150L / Q163K / T179S	93	97	-3
A44V / A71V / L124F / M150L / Q163K / T179S (mCherryR6)	81	89	-1

Peak emission wavelength, brightness, and thermostability resulting from reversion mutations to mCherry’s core.

## Conclusion

The successful designs reported here demonstrate that the problem of generating a monomeric RFP can be divided into a tractable computation-based surface design problem to achieve monomerization and a more challenging protein core optimization problem aimed at recovering and/or improving fluorescent properties. What is unclear is how surface modifications are coupled to the protein’s fluorescence properties, which are presumably controlled by details of the environment proximal to the chromophore in the core of the protein. We hypothesize that surface mutations leading to complete or partial monomerization perturb the structure (and/or stability) of the chromophore environment, requiring subsequent optimization to achieve desired fluorescent properties. This hypothesis is supported by the fact that previously reported RFP monomerization has involved significant mutation of the environment around the chromophore [18, 33–36]. Because DsRmCh has the optimized mCherry core, we were able to achieve monomerization that preserves fluorescent properties without the need for additional compensatory mutations. Designing the next generation of brighter and bathochromically shifted monomeric RFPs will thus require a more thorough understanding of the environment throughout the protein core and how mutation in the core can be used to prestabilize desired fluorescent properties.

## Supporting Information

S1 Fig Thermal Stability of Selected Variants. Selected DsRed, DsRmCh, and mCherry variants were purified and thermally denatured. The decrease in fluorescence as the proteins unfold was measured by quantitative real-time PCR, and shown here as normalized derivative curves, with the peak of each curve representing the apparent T_m_ of the protein. (A) The effects of tail deletions on mCherry and DsRed. (B) The stabilizing effect of the mCherry core in DsRmCh. (C) A representative mLib design variant is more thermostable than mCherry when mCherry’s tail is added back. (D) Core reversion mutations in mCherry destabilized the protein.
(TIFF)

S2 Fig Alignment of Previously Monomerized FPs. Alignment of ten previously monomerized FPs used to identify mutational hotspots in the directed evolution of FP monomers. Residues are numbered using DsRed numbering. Boxes indicate residues that were included in the mLib design, with shaded residues for each monomeric protein indicating that they were mutated during their evolution from a higher-order oligomeric parent. A small table at the bottom right indicates the parent protein of each monomeric variant in the alignment.
(TIFF)

S3 Fig mLib Variants. List of the 95 mLib variants. Each of the 17 designed positions is shown in a separate column. A “-”indicates no mutation from the reference wild-type DsRed sequence.
(TIFF)

S4 Fig Expression of mCherry Tail Variants. Expression levels for four mLib variants were measured in triplicate with either, no tail, a 5-residue DsRed tail (HHLFL), or an 11-residue mCherry tail (HSTGGMDELYK) measured as fluorescence in an induced bacterial culture (see Materials and Methods for protein expression details)
(TIFF)
