# Supplementary figures and images for: Computational Design of the β-Sheet Surface of a Red Fluorescent Protein Allows Control of Protein Oligomerization
Source: PLoS One. 2015 Jun 15;10(6):e0130582. doi: 10.1371/journal.pone.0130582 (PMC4468108; doi:10.1371/journal.pone.0130582)

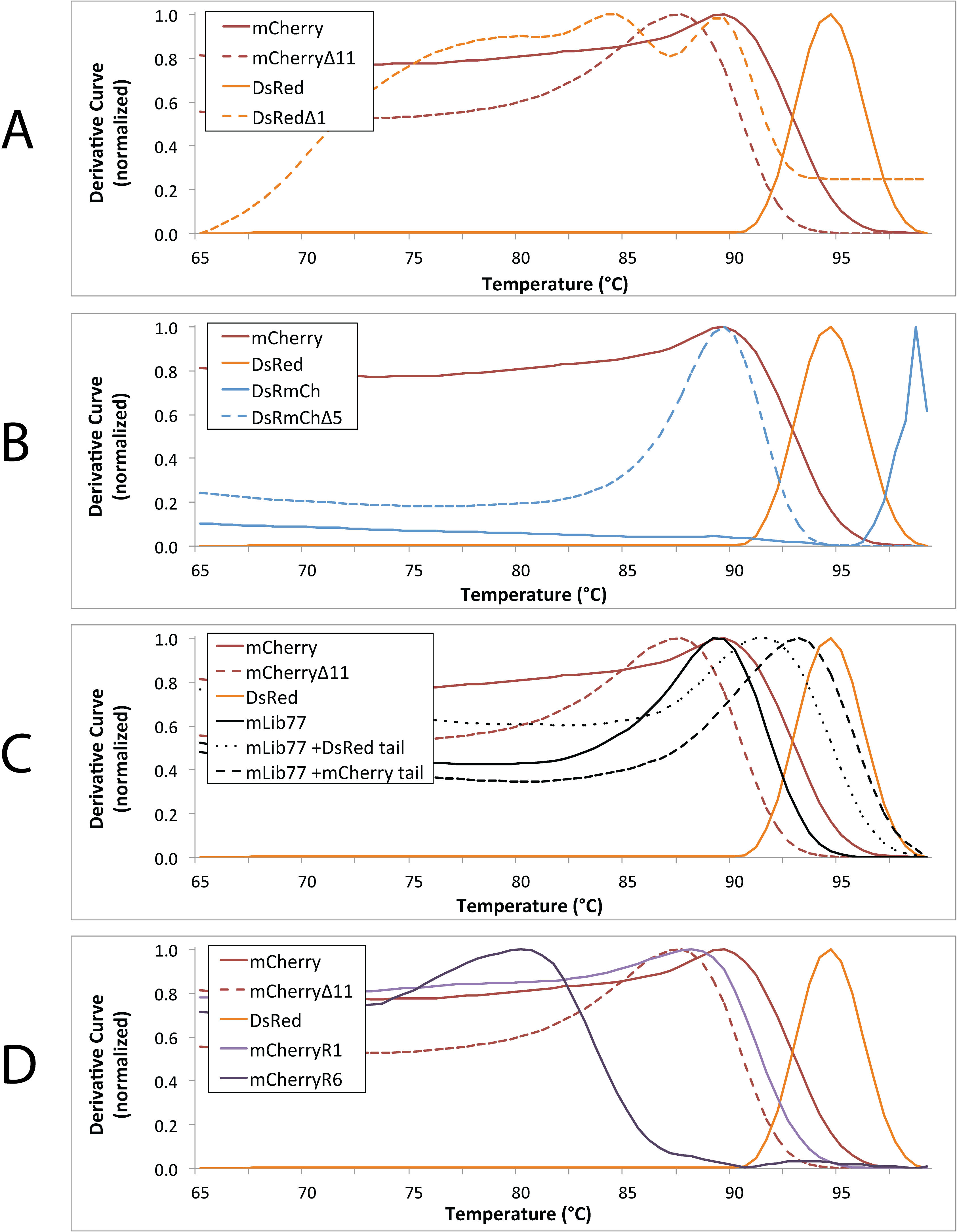

Supplement: S1 Fig — Selected DsRed, DsRmCh, and mCherry variants were purified and thermally denatured. The decrease in fluorescence as the proteins unfold was measured by quantitative real-time PCR, and shown here as normalized derivative curves, with the peak of each curve representing the apparent Tm of the protein. (A) The effects of tail deletions on mCherry and DsRed. (B) The stabilizing effect of the mCherry core in DsRmCh. (C) A representative mLib design variant is more thermostable than mCherry when mCherry’s tail is added back. (D) Core reversion mutations in mCherry destabilized the protein. (TIFF) [file pone.0130582.s001.tiff]

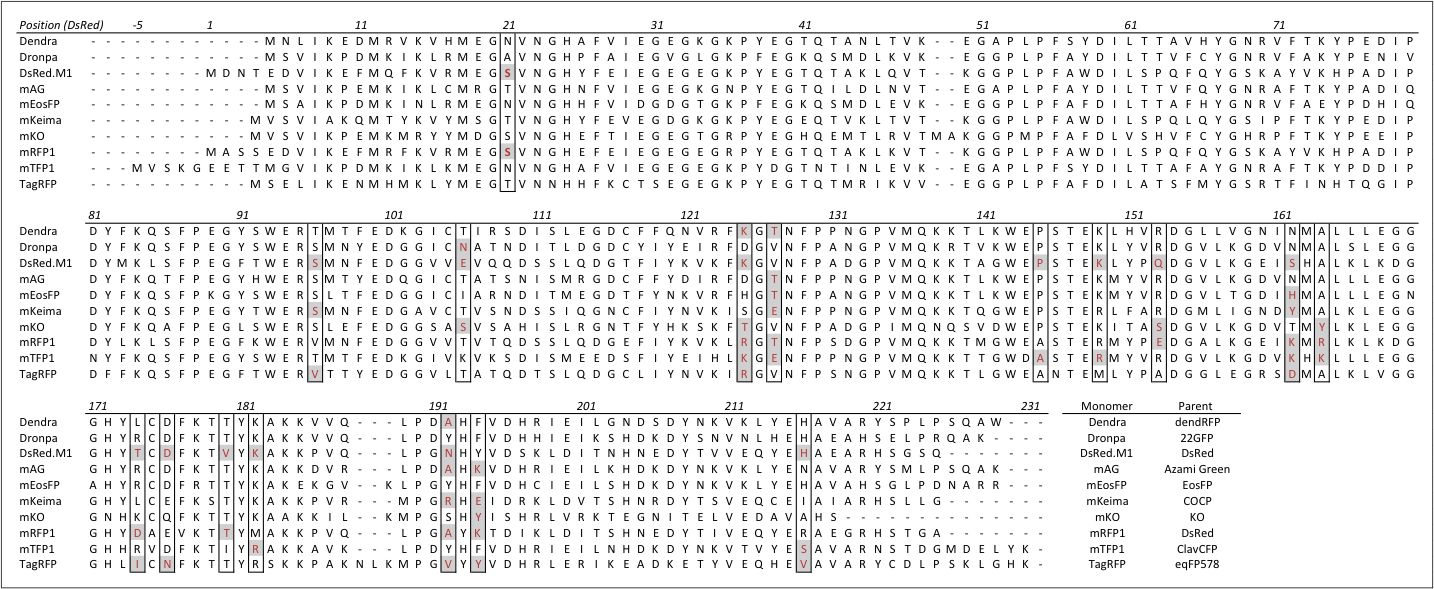

Supplement: S2 Fig — Alignment of ten previously monomerized FPs used to identify mutational hotspots in the directed evolution of FP monomers. Residues are numbered using DsRed numbering. Boxes indicate residues that were included in the mLib design, with shaded residues for each monomeric protein indicating that they were mutated during their evolution from a higher-order oligomeric parent. A small table at the bottom right indicates the parent protein of each monomeric variant in the alignment. (TIFF) [file pone.0130582.s002.tiff]

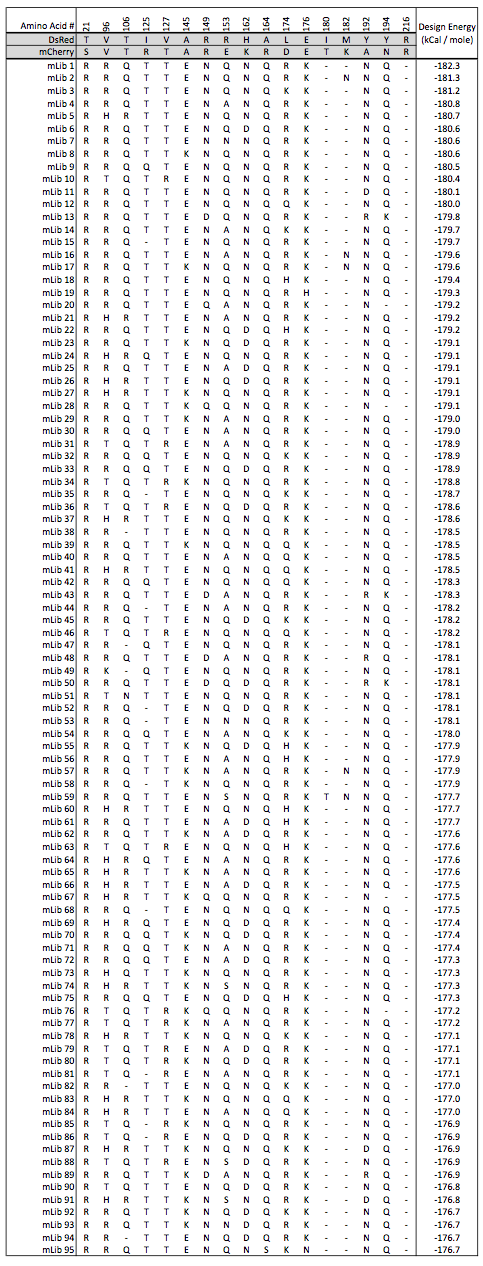

Supplement: S3 Fig — List of the 95 mLib variants. Each of the 17 designed positions is shown in a separate column. A “-”indicates no mutation from the reference wild-type DsRed sequence. (TIFF) [file pone.0130582.s003.tiff]

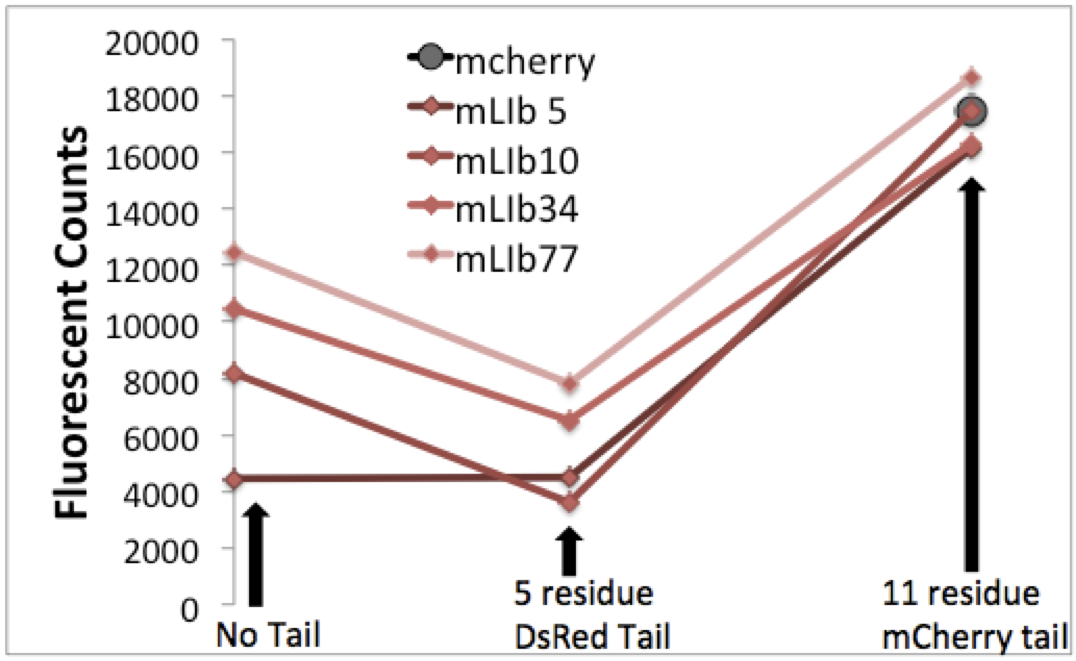

Supplement: S4 Fig — Expression levels for four mLib variants were measured in triplicate with either, no tail, a 5-residue DsRed tail (HHLFL), or an 11-residue mCherry tail (HSTGGMDELYK) measured as fluorescence in an induced bacterial culture (see Materials and Methods for protein expression details) (TIFF) [file pone.0130582.s004.tiff]
